# Photodynamic Therapy Directed to Melanoma Skin Cancer by Thermosensitive Hydrogel Containing Chlorophyll A

**DOI:** 10.3390/ph16121659

**Published:** 2023-11-29

**Authors:** Joabe Lima Araújo, Patrícia Bento da Silva, Bruno Fonseca-Santos, Sônia Nair Báo, Marlus Chorilli, Paulo Eduardo Narcizo de Souza, Luis Alexandre Muehlmann, Ricardo Bentes Azevedo

**Affiliations:** 1 Department of Genetics and Morphology, Institute of Biological Sciences, Darcy Ribeiro University Campus, University of Brasília, Brasília 70910-900, Brazil; 2 Department of Biotechnology, Health Sciences Institute, Federal University of Bahia, Salvador 40110-902, Brazil; bfonsecasantos@ufba.br; 3 Cellular Biology Department, Institute of Biological Sciences, Darcy Ribeiro University Campus, University of Brasília, Brasília 70910-900, Brazil; snbao@unb.br; 4 School of Pharmaceutical Sciences, São Paulo State University, Araraquara 14800-903, Brazil; marlus.chorilli@unesp.br; 5 Institute of Physics, Darcy Ribeiro University Campus, University of Brasília, Brasília 70910-900, Brazil; 6 Faculty of Ceilandia, University of Brasilia, Brasília 70910-900, Brazil

**Keywords:** photodynamic therapy, melanoma, skin cancer, hydrogel

## Abstract

Melanoma, a severe form of skin cancer intricately linked to genetic and environmental factors, is predicted to reach 100,000 new cases worldwide by 2040, underscoring the need for effective and safe treatment options. In this study, we assessed the efficacy of a photosensitizer called Chlorophyll A (Chl-A) incorporated into hydrogels (HGs) made of chitosan (CS) and poloxamer 407 (P407) for Photodynamic Therapy (PDT) against the murine melanoma cell line B16-F10. The HG was evaluated through various tests, including rheological studies, SEM, and ATR-FTIR, along with cell viability assays. The CS- and P407-based HGs effectively released Chl-A and possessed the necessary properties for topical application. The photodynamic activity of the HG containing Chl-A was evaluated in vitro, demonstrating high therapeutic potential, with an IC_50_ of 25.99 µM—an appealing result when compared to studies in the literature reporting an IC_50_ of 173.8 µM for cisplatin, used as a positive control drug. The developed formulation of CS and P407-based HG, serving as a thermosensitive system for topical applications, successfully controlled the release of Chl-A. In vitro cell studies associated with PDT exhibited potential against the melanoma cell line.

## 1. Introduction

Melanoma is an aggressive form of skin cancer in which genetic and environmental factors are closely involved in its development, progression, and malignancy [1,2]. There are four clinical presentations of melanomas: superficial spreading, nodular, lentigo maligna, and acral lentiginous. Superficialspreading is the most common clinical presentation. The incidence of melanoma worldwide has been steadily increasing, according to the latest report from the World Health Organization (WHO), with approximately 324,635 new cases of melanoma skin cancer reported globally in 2020 [3].

Melanoma is therapeutically resistant to conventional treatments such as chemotherapy (CT) and radiotherapy (RT), as well as to novel therapeutic approaches like immunochemotherapy and biochemotherapy [4,5]. Therefore, new therapeutic strategies have been explored against melanoma, including photodynamic therapy (PDT). PDT involves the application of a photosensitizer followed by exposure to light of a specific wavelength to generate reactive oxygen species (ROS) that can selectively damage tumor cells [6].

In the case of PDT against melanoma tumors, the selection of a photosensitizer is essential to achieve the desired photochemical action, capable of overcoming the melanin barrier presented by melanoma cells [4,7]. For this reason, we believe that Chlorophyll A (Chl-A) as a photosensitizer in PDT may exhibit robust photochemical activity due to favorable characteristics, such as the ability to absorb light in the red range (660 nm), allowing for greater penetration into the skin, as well as in the blue range (430 nm). Additionally, it has low toxicity and a significant capacity to generate reactive oxygen species (ROS) when activated by light. Moreover, recent studies by Liu et al. [8] investigated its cytotoxic action in assays against melanoma, where Chl-A demonstrated attractive cytotoxic activity in in vitro assays against the A375 melanoma cell line.

To achieve efficacy in protecting and delivering the photosensitizer to tumor cells, nanobiotechnological techniques, such as hydrogels (HGs), have been employed due to their favorable characteristics for drug delivery. These include biocompatibility, biodegradability, mechanical properties, and rapid responses to external stimuli [9]. HGs consist of three-dimensional networks of hydrophilic polymers that swell upon contact with water or biological fluids [10,11] and are classified as chemical or physical gels, depending on the synthesis method used.

The advantages of hydrogels (HGs) following external or natural stimuli allow for drug diffusion into the target tissue, making this technique appealing in biomedical applications. External stimuli, such as light irradiation, pH, magnetic fields, and temperature, can enable controlled drug release [9]. In this study, we explored the ability of the HG to be externally stimulated by an increase in temperature to release the photosensitizer Chl-A, which, upon exposure to light, will promote the production of reactive oxygen species (ROS), inducing the death of melanoma cancer cells. Thus, this study evaluated the anticancer activity of the photosensitizer Chl-A incorporated into hydrogels based on chitosan (CS) and poloxamer 407 (P407) applied in in vitro PDT against the murine melanoma cell line B16-F10.

## 2. Results and Discussion

### 2.1. Evaluation of Temperature T_sol-gel_ Properties of Formulations

**Figure 1:**
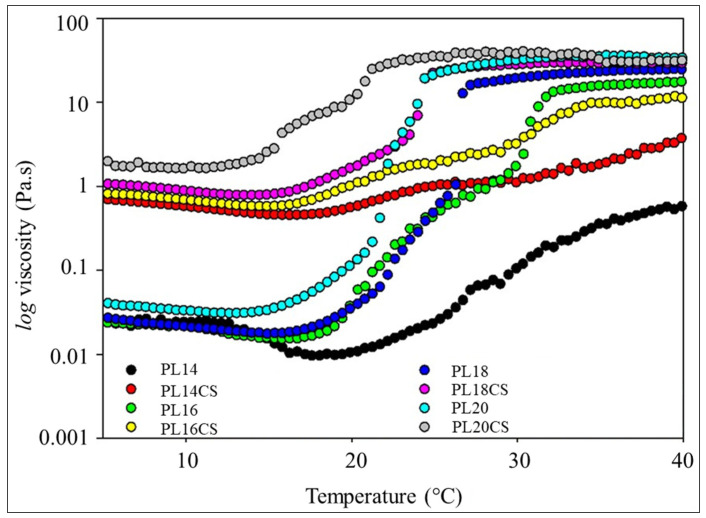
Temperature as function of viscosity of HGs samples. Note: On the X axis they represent the temperature range of the T_sol-gel_ phase and on the Y axis the viscosity information (Pa.s). Furthermore, the black, green, blue, and cyan color represent P407-only HGs at different concentrations, and the red, yellow, purple, and gray color represent CS-based HGs and P407 at different concentrations.

The temperature of the sol-gel transition (T_sol-gel_) of the HGs-CS-P407 was assessed at different concentrations through rheological analysis. It was observed that the formulation containing 16% of P407 yielded attractive T_sol-gel_ temperatures for topical application, with this phase change occurring in a range close to 32–34 °C, as depicted in Figure 1, aligning with the scientific literature on biomaterial formulations for topical applications [12]. Since thermosensitive HG formulations for topical applications should preferably have a T_sol-gel_ temperature close to the skin temperature of 34 °C [13], the temperature range of 32–34 °C facilitates the release of the active ingredient present in the gel by opening the mesh, prolonging the time the drug remains in the applied area due to reduced fluidity. Another important characteristic is that at temperatures below 32–34 °C, HGs behave like a liquid (solution), enabling easy application to the patient’s skin [12,13]. Additionally, formulations with T_sol-gel_ temperatures higher than body temperature (36.5 to 37.5 °C) will remain in a solution state, rendering the topical application of the biomaterial impractical [14].

Additionally, it is observed that the properties of CS stimulated an increase in the viscosity of P407 HGs. This allows us to infer that P407 HGs enhanced bioadhesive capacity and became more resistant when combined with CS, as shown in Table 1. Similar data were also obtained by Tuğcu-Demiṙöz [15]. Furthermore, it is noted that the higher the temperature, the higher the viscosity of the samples, favoring the use of HGs in topical applications at temperatures between 32 and 34 °C, thereby achieving greater resistance to the flow of the biomaterial, which reduces superficial spreading on the skin surface. In other words, the higher the viscosity of the HG, the less flow on the patient’s skin.

Another point to be discussed is the gelation time of the HGs. Formulations containing CS (1%) performed better in the T_sol-gel_ time phase compared to HGs with only P407, where only sample, V1.A, exhibited a gelation time of less than 40 s (Table 2). This indicates that the addition of CS reduces the gelation time of P407 hydrogels, accelerating the formation and association of micelles in the gel obtaining process. Characteristically, P407 gels have this gelation property in the T_sol-gel_ phase due to their negative solubility coefficient to form block copolymer micelles at a certain temperature. This thermogelation in the T_sol-gel_ phase can be explained through the hydrophobic interactions of copolymer chains, which combine into a micellar structure as the temperature increases [16].

The temperature rise induces the formation of micelles by desolvating the poly(propylene oxide) (PPO) block chains, leading to the creation of a void in the central region of the gel. It then also dehydrates the surface of the poly(ethylene oxide) (PEO) chains, causing these micelles to aggregate, forming a gel [17]. Therefore, the rapid transition of this gelation process is crucial to prevent leakage of the hydrogel in the initial moments of in situ application in the patient, avoiding the loss of essential biological components for treatment.

### 2.2. In Vitro Bioadhesion Assay

**Figure 2:**
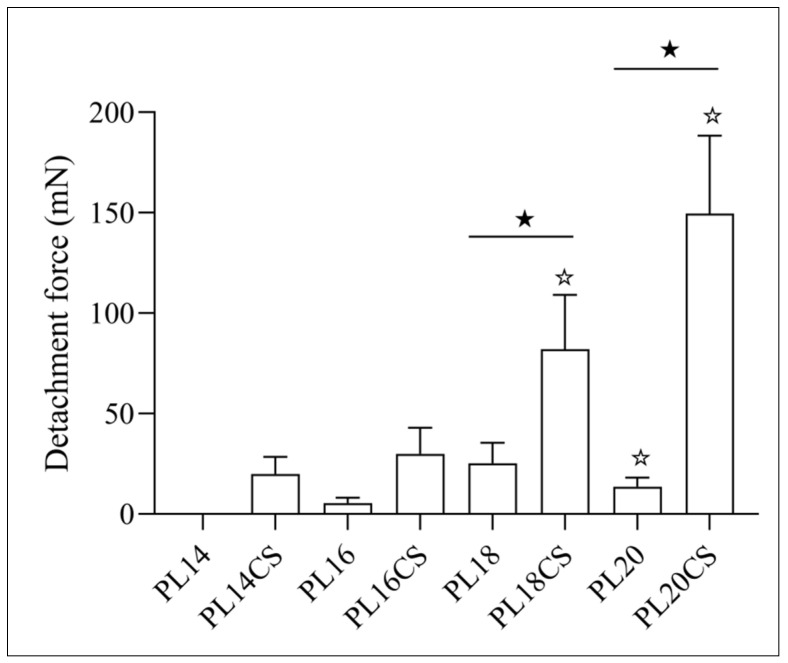
Bar graph of peel strength displayed by samples. Symbols: differences between groups, *p* < 0.05 (★); compared to PL18CS, *p* < 0.05 (☆); millinewton (mN).

The bioadhesive capacity was assessed in in vitro assays. Thus, HGs-CS-P407 and HGs-P407 at different concentrations achieved clinically attractive bioadhesion results, as observed in Figure 2, where the gel requiring the highest displacement force in millinewtons (mN) applied was the 20% P407 HG containing 1% CS (*w*/*v*). The second-best performance was obtained by the 18% P407 HG containing 1% CS (*w*/*v*), followed by the 16% P407 HG containing 1% CS (*w*/*v*), requiring a rupture force between the gel and the skin of 100 and 50 mN, respectively (Figure 2). This displacement force is the applied force needed to break the fluidity of a biomaterial under shear stress.

This evaluation is essential because bioadhesive systems, such as drug carriers, have the ability to prolong the release time of the active principle at the site of application, which favors the permanence of the drug in the lesion for a longer time, reducing the frequency of new gel applications in the patient, in addition to facilitating patient adherence to treatment [18]. Therefore, it becomes an attractive biomaterial in dermal applications, as other biomaterials such as creams, solutions, and lotions do not have a prolonged effect after topical application, since they can be easily removed by the patient’s physical movements, humidity, and temperature [19].

It is noteworthy that HGs containing CS have greater bioadhesive capacity than HGs containing only P407, which shows that the increase in bioadhesion is related to the addition of CS in the gel formulation. It is observed that the CS gave a higher response, in relation to both the bioadhesion and detachment force. However, the free gel shows a considerable rate of detachment strength, in which, as there is a minimal increase in the concentration of P407 in the HG (free), there is an increase in bioadhesion, as observed in PL14, PL16, and PL18 (Figure 2), and PL20 showed a lower detachment force than PL18, even with its higher concentration of P407. This shows that intermediate concentrations (14, 16, and 18%) of P407 cause a significant increase in bioadhesion, and on the contrary, that with higher concentrations (20%) of P407, there is no increase in bioadhesion. These results agree with studies carried out by Cafaggi et al. [20].

Based on the above, it is observed that the P407 gel has bioadhesive properties regardless of high concentrations of poloxamer, and that both CS and P407 act in the formation of the bioadhesive matrix [20] because this phenomenon occurs by numerous steps, such as hydration of the polymer, the interpenetration of the bioadhesive polymer in the gel, and the swelling of the meshes [21]. Thus, CS provides adhesive properties that maximize contact with the skin mucosa and after absorbing moisture that allow for polymer interpenetration and subsequently hydrogen bonding. It is suggested through these results that the combination of P407 copolymer with CS resulted in a gel with promising bioadhesive properties, in addition to flexibility and mobility that promote greater interpenetration in the skin mucosa.

### 2.3. Characterization by ATR-FTIR

**Figure 3:**
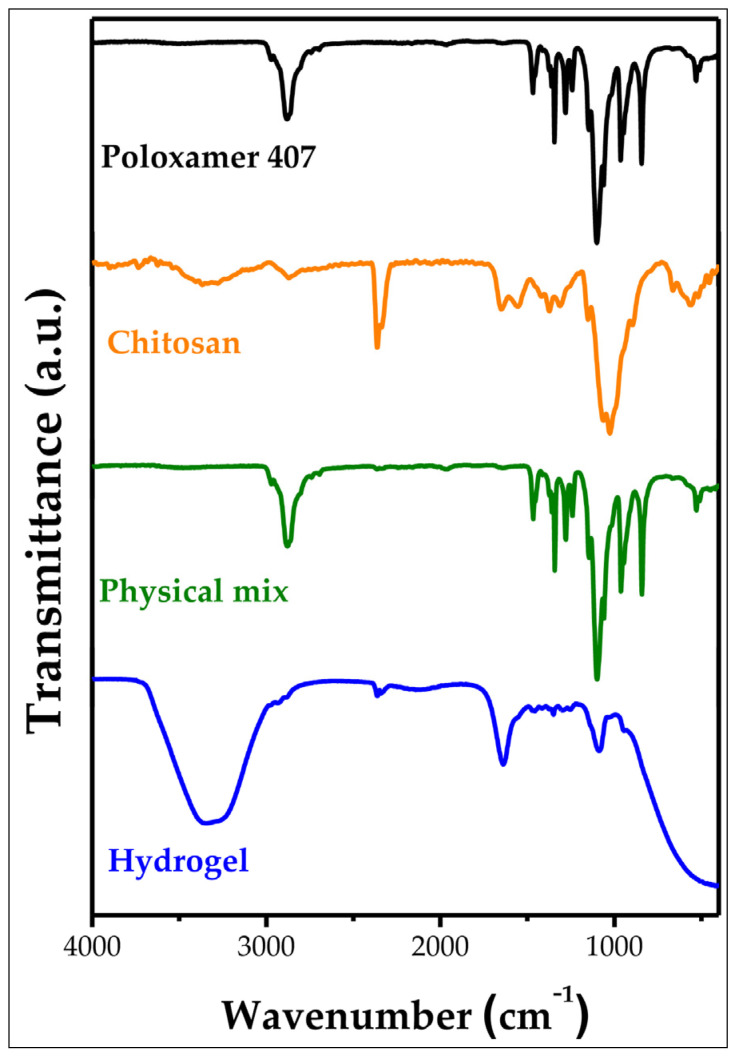
Vibrational spectrum in the infrared region (400–4000 cm^−1^) of poloxamer 407, chitosan, physical mixture, and hydrogel.

After the rheological analysis, the PL16CS sample (P407 hydrogel containing 16% CS), which showed better results for topical application, was submitted to ATR-FTIR analysis. The interaction of functional groups of the polymers used to compose PL16CS was studied by ATR. Figure 3 shows the vibrational spectra of the hydrogel constituents (PL16CS), the physical mixture and the separate components (P407 and CS).

The FTIR spectrum of P407 (Figure 3, black line) is characterized by the main absorption peaks at 2877 cm^−1^ of the aliphatic C-H group. The bands at 1469 cm^−1^ and 1100 cm^−1^ confirm the presence of C-O groups of ether [22]. Moreover, a band is also observed at 1340 cm^−1^, which is assigned to the deformation of O-H in the plane.

In the CS spectrum (Figure 3, orange line), a broad band of medium intensity is observed in the region of 3539–3110 cm^−1^, which corresponds to N-H and O-H stretching, in addition to intramolecular hydrogen bonds. The widened band near 2873 cm^−1^ can be attributed to the asymmetric stretching of the C-H. The presence of the bands at 1658 cm^−1^ and 1555 cm^−1^ are attributed to δN-H. The scissor-like deformation for CH_2_ and the symmetrical stretching of CH3 was confirmed by the presence of bands close to 1420 cm^−1^ and 1376 cm^−1^, respectively. The absorption band at 1150 cm^−1^ can be attributed to the asymmetric ν bridged by C-O-C. The absorption bands at 1062 cm^−1^ and 1025 cm^−1^ correspond to νC-O. All bands observed in the spectrum of the CS sample were also reported by other authors [23,24]. The peak at 897 cm^−1^ corresponds to the out-of-plane deformation of the C-H of the monosaccharide ring. The bands at 2359 cm^−1^ and 2335 cm^−1^ are attributed to the asymmetric and symmetric stretching vibration of the CO_2_ present during the measurement [25].

It is observed in the FTIR spectrum of the physical mixture, Figure 3 (green line), that the presence of both bands areattributed to CS and P407. The bands at 2971 cm^−1^ and 2878 cm^−1^ are attributed to symmetric and asymmetric νC-H, respectively, of the CH_2_ and CH_3_ present in the P407 and CS structures. The presence of the low-intensity band at 1649 cm^−1^ is attributed to the δN-H band present in the CS. The bands present at 1100 cm^−1^ and 1060 cm^−1^ can be attributed to νC-O and confirm the presence of C-O-C groups characteristic of ether in P407, as well as in CS. It was not possible to observe significant shifts in the bands of the physical mixture, which allows for the inference that there was only a physical mixture of the components, which was expected, since in this sample, no water was added, and the entire HG preparation process was not performed.

In the hydrogel spectrum, Figure 3 (blue line), an intense and broad band at 3328 cm^−1^ can be observed, which can be attributed to δN-H and νO-H, and to hydrogen bonds, which characterizes a very broad band [26]. These hydrogen bonds can come from the hydroxyl group of P407 with the N of the NH group of CS. For example, the low-intensity bands at 2927 cm^−1^ and 2879 cm^−1^ that are characteristic of symmetrical and asymmetrical stretching of both P407 and CS are observed in the spectrum. In the spectrum, a band of medium intensity extended at 1642 cm^−1^ can also be observed, which can be attributed to the δN-H band present in chitosan, and this is displaced by 16 cm^−1^ when compared to the CS spectrum, which it is a strong indication that the hydrogel is having an interaction via the -NH_2_ group of CS. Giventhe alterations observed in the characteristic bands of P407 as well as of CS in the hydrogel spectrum that was mentioned above when compared to the isolated P407 and CS spectra, it is suggested that this hydrogel is formed by hydrogen-type interactions between the H of the -OH group present in P407 with the N of the -NH_2_ group of the CS [27]. Furthermore, no contaminating compounds were identified, which suggests the purity of the formulation.

### 2.4. Characterization by SEM

**Figure 4:**
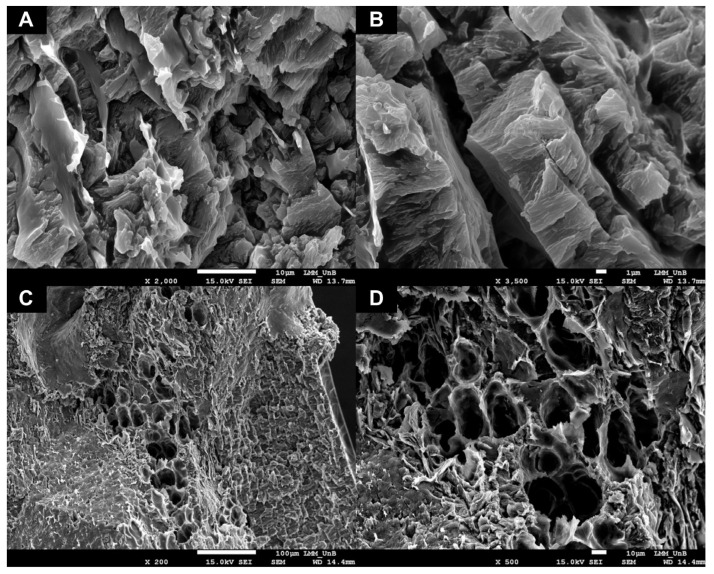
Surface morphology of the hydrogel based on chitosan and poloxamer 407. Note: (**A**) We observe a porous, rough surface structure with closed meshes. (**B**) It is observed with a 3500× zoom for better visualization of the surface structure. (**C**) We observe a porous structure in the form of alveoli through which the drug is released when HG-CS-P407 enters the sol-gel transition phase. (**D**) It is observed with a 500× zoom for better visualization.

According to the characteristics elucidated in the rheological studies addressed in Section 2.1 and Section 2.2, it was possible to visually confirm the presence of a porous and rough structure in its dispersed state through the SEM technique (Figure 4A,B). Furthermore, it can be observed that the internal transversal structures exhibit a set of alveolar pores (Figure 4C,D).

These surface morphological structures are specific characteristics in HG formulations, as reported in studies by Kim and Chu [28], who investigated SEM characterization techniques of methacrylate–dextran HGs. In those studies, they observed that HGs had different characteristics when they were in open and closed loops. When in an open loop, it presented a porous and rough structure, similar to that observed in this study with HG-CS-P407, in addition to having a non-porous surface and transversal structure when the loops were closed.

Diniz et al. [29] also reported that the Pluronic F-127 HG had a tubular aspect, similar to alveoli, in addition to presenting reticular networks similar to those obtained in our studies with HG-CS-P407. The authors also reported that these alveoli are essential for the release and delivery of chemical compounds with biological activity and that the crosslinking and swelling of the HG induce morphological changes that can either increase or decrease the diffusion and permeation of biomaterials.

Another point to be highlighted is that the rapid degradation of biomaterials is unfavorable in topical applications, in which more mature biomaterials can be advantageous in this sense [30]. In these results, through SEM, we were able to observe greater maturity in relation to the degradability of the biomaterial, confirming the rheological results of bioadhesion discussed in previous sections and the optimization of both the mechanisms of sustained release of the drug and its morphological preservation for a longer time. These characteristics were not obtained in the studies by Diniz et al. [29], who formulated the hydrogel only with P407.

### 2.5. Biological Assay

**Figure 5:**
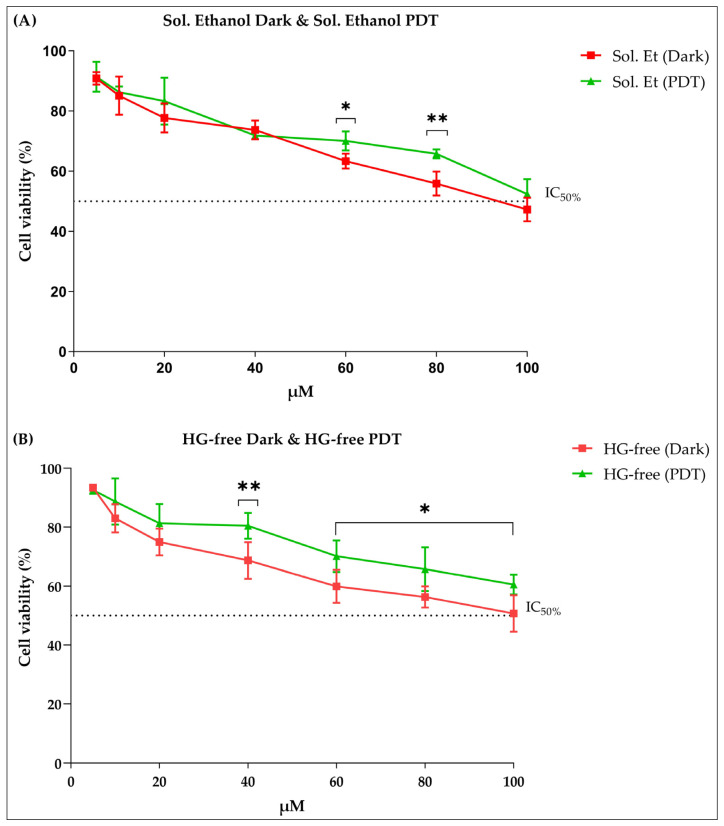
Cytotoxic activity of ethanol and HG-free solutions against melanoma cells. (**A**) In the red line, we have the ethanol solution in the dark, and in the green line, we have the ethanol solution exposed to PDT. (**B**) In the red line, we have the free hydrogel in the dark, and in the green line, we have the free hydrogel exposed to PDT. Note: Sol. Et, ethanol solution; HG-free, hydrogel; PDT, photodynamic therapy; µM, micromolar; %, percentage; IC_50_, mean inhibitory concentration. Values with *p* ≤ 0.05 were considered statistically different. Asterisks represent the level of statistical difference between sample results. The significance level is 95%. The more asterisks there are, it means that the statistical difference is greater between the sample results.

The PL16CS formulation (P407 hydrogel containing 16% CS) with the best characteristics for topical application was selected to receive Chl-A, in which its cytotoxic activity against B16-F10 murine melanoma cells was evaluated after PDT. As expected, ethanol was cytotoxic to B16-F10 cells, though the cells presented more than 50% viability even at the highest concentration even at the highest concentration of ethanol (Figure 5A). When the ethanol solution (2%) is kept in the dark, a result similar to that obtained with the ethanol solution (2%) excited by LED is observed, as shown in Figure 5A. Statistical differences between cells in the dark and exposed to LED light were observed only for the concentrations of Chl-A of 60 and 80 µM.

It is possible to observe a statistical difference in the concentrations of 60 and 80 µM of Sol. Et when comparing the two types of treatments, PDT and dark. This low reduction in cell viability is not related to exposure to LED, considering that cell viability is higher when cells were exposed to PDT (Figure 5A), which was also observed in studies by Shah et al. [31], in which they evaluated the photodynamic activity of four types of LEDs when cancer cells were exposed to them. Furthermore, it was observed that LEDs at wavelengths of 630 nm (red), 515 nm (green), and 410–456 nm (blue) did not influence the reduction in cell viability.

The same behavior is also observed in Figure 5B with cells subjected to the HG-free treatment. There is a statistical difference in the concentrations of 40, 60, 80, and 100 µM, demonstrating that the exposure of cancer cells to LED does not influence the reduction in cell viability. A cytotoxic action of the controls (solution ethanol and HG-free) is evidenced, but it is not significant since this action at the highest concentration (100 µM of solution ethanol and HG-free) does not inhibit the viability of the cancer cells by 50% when exposed to PDT (Figure 5A,B).

**Figure 6:**
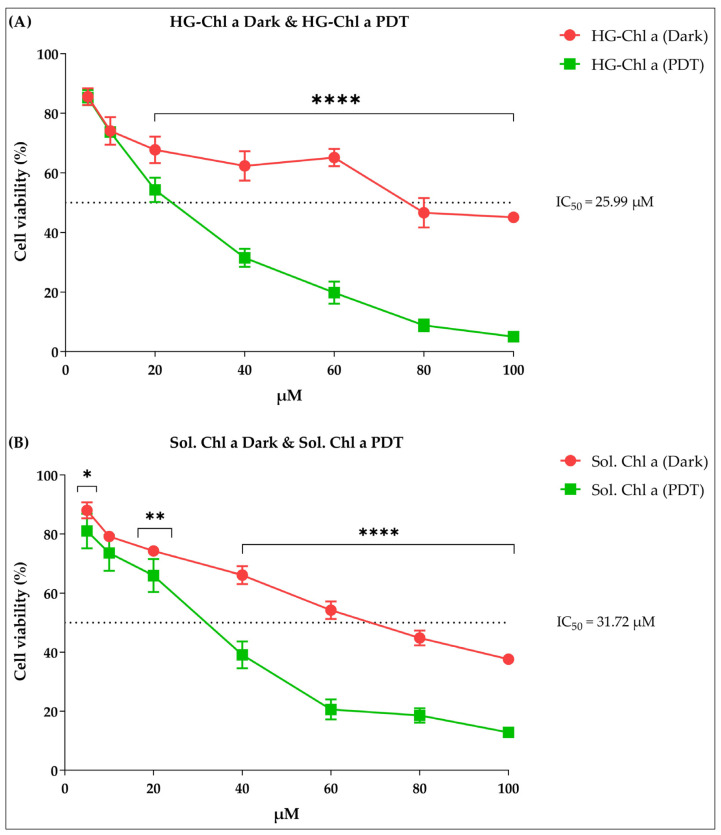
Cytotoxic activity of HGs containing Chl-A and Chl-A solutions against melanoma cells. (**A**) In the red line, we have the hydrogel containing Chlorophyll A in the dark, and in the green line, we have the hydrogel containing Chlorophyll A exposed to PDT. (**B**) In the red line, we have Chlorophyll A solution in the dark, and in the green line, we have Chlorophyll A solution exposed to PDT. Note: HG-Chl-A, hydrogelcontaining Chlorophyll A; HG-free, hydrogelfree; PDT, photodynamic therapy; µM, micromolar; %, percentage; IC_50_mean inhibitory concentration. Values with *p* ≤ 0.05 were considered statistically different. Asterisks represent the level of statistical difference between sample results. The significance level is 95%. The more asterisks there are, it means that the statistical difference is greater between the sample results.

When the cytotoxic activity of HG containing Chl-A (HG-Chl-A) against melanoma cells was evaluated, a high inhibitory action on cell viability was observed in both treatments, dark and PDT (Figure 6A). However, this inhibitory action was more intense when melanoma cells were treated with HG-Chl-A and irradiated with LED, having an IC_50_ of 25.99 µM, a concentration well below the IC_50_ of 173.8 µM of CDDP, for example [32].

Furthermore, there is a statistical difference in the tested concentrations (20–100 µM) when comparing dark versus PDT treatments, as illustrated in Figure 6A. Given these results, it is possible to infer that the promoted cytotoxic action occurs from the excitation of photosensitizer Chl-A by light, which stimulates the formation of ROS and induces cell death in melanoma cells. Therefore, this activation of the photosensitizer to its triplet excited state allows it to interact with different surrounding molecules (type I photoreaction) or react with molecular oxygen (type II photoreaction) [33], in which the photodynamic action will act on melanoma cells, damaging their components and inducing cell death by apoptosis, necrosis, and/or autophagy [34,35]. It is also observed that this cytotoxic action against melanoma cells is closely related to the concentration administered; the higher the concentration used, the greater the rate of inhibition of cell viability.

Regarding the results obtained with the Chl-A solution against melanoma cells, both in the dark and in the treatment with PDT in vitro, it is observed that Chl-A presents cytotoxic activity. However, the cytotoxic activity of the Chl-A solution in the dark is low compared to the cytotoxic activity employed by Chl-A solution excited by LED. As seen in Figure 6B, the photodynamic effect is evidenced by the fact that the solution of Chl-A in the dark had an IC_50_ above 70 µM against B16-F10 cells, while this solution excited by LED gave an IC_50_ of 31.72 µM (Figure 6B). This shows that Chl-A is a good photosensitizer in PDT with agreat potential in the medical field, as also highlighted by Gray and Fullarton [36]. Minimally invasive therapies such as PDT are crucial for the medical evolution of skin cancer treatments. The effectiveness of this therapy requires a photosensitizer with potential photocytotoxic activity, which inhibits cancer cells and provides good adherence from the patient to the treatment.

It was also observed that when Chl-A was incorporated into the HG, there was a slow release of the photosensitizer, and this release allowed for the maintenance of the activity in melanoma cells, decreasing cell viability as the concentrations increased, as seen in Figure 6A for HG-Chl-A (PDT). On the other hand, in Figure 6B, an opposite behavior of Sol. is observed Chl-A (PDT), in which, upon reaching a concentration of 60 µM, cell viability tended to increase. This effect can occur when there is no controlled release of the drug, where in these cases, the loss of pharmacological activity under the lesion is rapid, while controlled drug delivery systems do not immediately release all the drug, instead doing so gradually and continuously over time at different times and places, which increases the effectiveness of the treatment and increases the time of pharmacological action under the lesion [9].

From the above, it can be inferred that the thermosensitive system adopted in this study provides a controlled release of photosensitizer Chl-A in PDT in vitro. However, controlled drug release studies are needed to obtain conclusive results regarding this evidence.

## 3. Materials and Methods

### 3.1. Materials and Reagents

Tripan blue, sodium bicarbonate, Chl-A of spinach, DMEM, P407 (Pluronic^®^ F-127), low-molecular-weight CS were purchased from Sigma Aldrich (St. Louis, MO, USA). Penicillin and streptomycin, bovine fetal serum, and tripsine-EDTA were acquired from Gibco (Waltham, MA, USA). MTT was purchased from Invitrogen (Waltham, MA, USA). Other materials and reagents used in this study were analytical grade and/or suitable for use on cells culture.

### 3.2. Formulation of HGs and Incorporation of Chl-A in HGs

The HGs based on CS and P407 (HG-CS-P407) were prepared according to the protocol by Gratieri et al. [37], with some modifications. Briefly, low-molecular-weight CS dispersions with a concentration of 1.0% (*w*/*v*) were prepared by dissolving CS in 1.0% acetic acid solution (*v*/*v*) under constant stirring for approximately 3 h until complete homogenization. Then, the pH of the CS dispersions was adjusted to a pH between 6.0 and 6.5 with 10 M NaOH. For loading, separately, Chl-A was dissolved in 1 mL of 1% ethanol (*v*/*v*) with the aid of an ultrasonic bath (Q5.9/40, Ultronique Eco-Sonics, Newcastle, DL, USA). Subsequently, Chl-A was added dropwise under constant stirring in the CS dispersion in an environment without light. After this step, the CS dispersions containing Chl-A were kept in an ice bath under constant agitation, for the addition of different P407 concentrations. After the complete homogenization of P407, the HG-CS-P407 was kept at rest in a refrigerator at a temperature of 4 °C, for at least 24 h until no more bubbles were observed in the gel. The compositions of HG-CS-P407 are shown in Table 3. To blank formulations, the loading procedure was suppressed.

### 3.3. Evaluation of the Sol-Gel Transition Temperature (T_sol-gel_) of HG-CS-P407

The evaluation of the temperature of the T_sol-gel_ was carried out in a hybrid rheometer model HDR-1 (TA. Instruments, Newcastle, DL, USA) attached to a plate with a diameter of 40 mm. Prior to analysis, samples were placed in an ice bath, and the bottom plate temperature was adjusted to 5 °C. The samples were gently placed evenly on the bottom plate with the aid of a plastic pipette, and the top plate was lowered to contact the sample at a height of 500 µm. The samples were acclimatized to a temperature of 5 °C for 3 min (min), and then the test was initiated. The viscosity of the samples was evaluated at a shear rate of 10 s^−1^, and a temperature ramp of 5 °C/min in the range of 5–40 °C.

### 3.4. Evaluation of T_sol-gel_ Time

The determination of the T_sol-gel_ time was also verified by the test tube inclination method. 2.0 mL of the formulations of HG-CS-P407 and HG-P407 (see Table 3) were transferred to a test tube and kept in a water bath at 34 °C. Every 5.0 s, the test tube was removed from the water bath and tilted at an angle above 180° to observe the state of the fluidity of the samples. If the T_sol-gel_ phase was not observed after 40 s, the formulations were considered unfeasible for the use in topical application. Gelling time was recorded when there was no fluidity of HG-CS-P407 and/or HG-P407 in the test tube and it characterizes the change from the solution phase to the gel phase [38,39].

### 3.5. In Vitro Bioadhesion Assay

Fresh ears from 6-month-old pigs were obtained from a local slaughterhouse and prepared according to the method described by Dick and Scott [40]. Ears were cleaned with water (25 ± 0.5 °C), and those with lesions were discarded. The undamaged skins were removed from the cartilage with a scalpel and the layer containing the stratum corneum and the epidermis was separated from the adipose tissue using a dermatome TCM 300 (Nouvag, Goldach, Switzerland) with a thickness of 400 µm and was frozen at −20 °C for up to 4 weeks.

On the day of the experiment, the skins were thawed in a physiological saline solution, at 32 ± 0.5 °C for 30 min; A TA-XTplus texture analyzer (Stable Micro Systems, Surrey, UK) containing a cylindrical probe (diameter 10 mm) was used to assess peel strength. The cylindrical probe was covered with masking tape 101LA (3M^®^), and two drops of a cyanoacrylate-based adhesive (Loctite^®^ Super Bonder^®^) were added, both purchased at a local supermarket, São Paulo, Brazil. Then, the skinswereattached to the lower end of the cylindrical probe, and the edges secured with a rubber ring. The HG-CS-P407 and HG-P407 were placed below the probe and immersed in a water bath at 32 ± 0.5 °C.

The test was performed by lowering the probe at a constant speed (1 mm/s) until the skin and sample made contact, detected by a trigger force of 2 mN. The skin and the sample were kept in contact for 60 s, and no force was applied during this interval. After 60 s, the skin was pulled upwards (0.5 mm/s) until the contact between the surfaces was broken. During the experiment, a force–time curve was recorded from which the detachment peak was calculated. Five replicates were analyzed at 32 ± 0.5 °C.

### 3.6. Attenuated Total Reflectance with Fourier Transform Infrared Spectroscopy (ATR/FTIR)

In this study, spectroscopy in the infrared region was used to determine the interaction of the functional groups of the polymers used to compose the HGs. The technique was performed using a Bruker Vertex 70 equipment (Bruker Optik, Ettlingen, Germany) with attenuated total reflectance (ATR), with a spectral resolution of 4 cm^−1^ and 96 scans in the range of 400–4000 cm^−1^. The isolated components (P407 and CS), the physical mixture (pm), and the most attractive HG formulation after rheological analysis were evaluated. This study also served to assess the purity of HG, identifying any contaminating compound in the sample.

### 3.7. Scanning Electron Microscopy (SEM)

The morphology of HG-CS-P407 were analyzed using Scanning Electron Microscopy (SEM), as follows in studies by Zhao et al. [41]. HG-CS-P407 was frozen with liquid nitrogen for 15 min. Then, it was lyophilized for 24 h in the Speed Vac equipment. Then, fractures were performed in the material followed by metallization with gold in the Leica EM SCD 500 equipment. The analysis was performed using SEM (JEOL JSM-7001F).

### 3.8. Cell Culture Conditions

The B16-F10 murine melanoma cell line was purchased from the Rio de Janeiro Cell Bank (BCRJ, Rio de Janeiro, RJ, Brazil). Cell culture of B16-F10 was performed in DMEM medium (Dulbecco’s Modified Eagle Medium) supplemented with 10% fetal bovine serum (FBS) and antibiotics (penicillin (100 μg/mL) and streptomycin (100 μg/mL)) at 37°C in 5% CO_2_ in a cell incubator.

### 3.9. PDT Mediated by HG-CS-P407 Containing Chl-A

This study was based on the protocol by Morais et al. [32]. 96-well microplates were used to culture B16-F10 cells (1 × 10^4^ per well), and the cells were exposed to samples of HGs-CS-P407 and HGs-CS-P407-Chl-A, in addition to the respective controls: Chl-A solution in ethanol (2%) and ethanol solution at concentrations of 20, 40, 60, 80, and 100 µM, respectively, for approximately 4 h. After that, the medium containing HG-CS-P407- Chl-A was discarded, and the cells were washed twice with PBS. Finally, the cells were irradiated with a light emitting diode (LED) (430 nm at 27 J/cm^2^) for approximately 15 min. After irradiation, cells were kept in the dark for 24 h in an incubator containing 5% of CO_2_.

The choice of the 430 nm wavelength to excite the chlorophyll molecule in PDT was based on the molecule’s ability to possess two high-absorption wavelength regions, at 430 nm (Soret band) and 660 nm (Q band). In this study, we evaluated the efficiency of the Soret band (430 nm), where the literature still reports the chlorophyll’s capacity to produce a high yield of singlet oxygen (^1^O_2_) [42]. These are highly toxic species responsible for the death of cancer cells via the type II photochemical mechanism (energy transfer from the photocatalyst in its excited triplet state to ^3^O_2_), which is considered the main pathway of photodynamic activity [43].

### 3.10. Cell Viability Assays

Cell viability experiments were conducted by MTT assay (3-(4,5-dimethylthiazol-2-yl)-2,5-diphenyltetrazolium bromide). Briefly, after 24 h of PDT treatment, 96-well microplates containing B16-F10 cells (1 × 10^4^ per well) received MTT at 0.5 mg/mL. After that, the microplates were incubated for 2 h at a temperature of 37 °C and 5% CO_2_. Then, the supernatant was removed, and the dark blue formazan crystal was dissolved with DMSO. The reading of the microplates was carried out by a spectrophotometer at an absorbance of 595 nm, and the results were expressed in percentage with the control. Each treatment was performed in triplicate and repeated in three independent experiments.

### 3.11. Statistical Analysis

The results obtained in the HGs-CS-P407-Chl-A formulation analysis were submitted to be evaluated by the unpaired t-test using GraphPad Prism Software, v9.5.0, La Jolla, CA, USA, in which values with *p* ≤ 0.05 were considered statistically different.

## 4. Conclusions

The CS- and P407-based HG formulation proved effective in releasing the photosensitizer Chl-A. Additionally, it exhibited essential characteristics for topical applications, such as a temperature of 32 °C for the T_sol-gel_ phase, a time of 10.20 s for the T_sol-gel_ phase, and a displacement force of 50 mN. All these properties favored the drug’s bioavailability, controlled release of the active ingredient, and increased residence time of the photosensitizer at the applied site due to its low fluidity. Moreover, it demonstrated efficacy in in vitro PDT, where the photosensitizer exhibited high photodynamic activity with an IC_50_ of 25.99 µM, a significant reduction in the cell viability of melanoma cells compared to the IC_50_ of 173.8 µM for CDDP, as reported in literature studies. Therefore, it was possible to demonstrate the effectiveness of the CS and P407-based HG formulation as a thermosensitive system in topical applications for the release of the photosensitizer Chl-A in PDT against melanoma.

## Figures and Tables

**Table 1:** Data of the T_sol-gel_ temperature and viscosities of the hydrogels.

Sample	Viscosity at 5 °C (Pa.s)	Viscosity at 20 °C (Pa.s)	Viscosity at 34 °C (Pa.s)	Transition Temperature (°C)
PL14	0.02	0.01	0.02	24.50
PL14CS	0.69	0.58	1.63	18.10
PL16	0.02	0.04	13.25	19.0–31.74
PL16CS	0.81	1.01	6.21	16.73–34.46
PL18	0.03	0.04	21.31	18.6–26.70
PL18CS	1.07	1.64	29.44	15.81–24.20
PL20	0.04	0.11	34.28	14.43–24.90
PL20CS	1.75	10.59	35.11	14.44–22.64

Note: (PL14) 14% poloxamer 407 hydrogel; (PL14CS) 14% poloxamer 407 hydrogelcontaining 1% chitosan; (PL16) 16% poloxamer 407 hydrogel; (PL16CS) 16% poloxamer 407 hydrogelcontaining 1% chitosan; (PL18) 18% poloxamer 407 hydrogel; (PL18CS) 18% poloxamer 407 hydrogelcontaining 1% chitosan; and (PL20) 20% poloxamer 407 hydrogel; (PL20CS) 20% poloxamer 407 hydrogelcontaining 1% chitosan.

**Table 2:** Time of T_sol-gel_ phase of HG-CS-P407 and HGs-P407.

Identification	CS (%)	P407 (%)	Time (s)
V1	1.0	14	10.24
V2	1.0	16	10.20
V3	1.0	18	10.23
V4	1.0	20	n/a
V1.A	n/a	14	30.12
V2.B	n/a	16	n/a
V3.C	n/a	18	n/a
V4.D	n/a	20	n/a

Note: CS, chitosan; P407, poloxamer 407; V1, vial 1; V2, vial 2; V3, vial 3; V4, vial 4; V1.A, vial 1A; V2.B, vial 2B; V3.C, vial 3C; V4.D, vial 4D; n/a, not applicable. Formulations V4, V2.B, V3.C, and V4.D received the term “n/a” because they did not reach the T_sol-gel_ temperature after 40 s of exposing the samples to a temperature of 34 °C. Therefore, they are unfeasible formulations for topical application.

**Table 3:** Compositions and concentrations of HG-CS-P407 formulations.

Identification	CS (%)	P407 (%)
V1	1.0	14
V2	1.0	16
V3	1.0	18
V4	1.0	20
V1.A	n/a	14
V2.B	n/a	16
V3.C	n/a	18
V4.D	n/a	20

Note: CS, chitosan; P407, poloxamer 407; V1, vial 1; V2, vial 2; V3, vial 3; V4, vial 4; V1.A, vial 1A; V2.B, vial 2B; V3.C, vial 3C; V.4D, vial 4D; n/a, not applicable.

## Data Availability

Data are contained within the article.
